# Biosensors for the Isolation and Detection of Circulating Tumor Cells (CTCs) in Point-of-Care Settings

**DOI:** 10.3390/mi14051035

**Published:** 2023-05-12

**Authors:** Isaac Goldstein, Sobia Alyas, Waseem Asghar, Azhar Ilyas

**Affiliations:** 1 Bio-Nanotechnology and Biomaterials (BNB) Lab, New York Institute of Technology, Old Westbury, NY 11568, USA; 2 Paul D. Schreiber High School, Port Washington, NY 11050, USA; 3 Institute of Molecular Biology and Biotechnology (IMBB), The University of Lahore, Lahore 54000, Pakistan; sobia.alyas@imbb.uol.edu.pk; 4 Department of Electrical Engineering and Computer Science, Florida Atlantic University, Boca Raton, FL 33431, USA; wasghar@fau.edu; 5 Department of Electrical and Computer Engineering, New York Institute of Technology, Old Westbury, NY 11568, USA

**Keywords:** circulating tumor cells, immunoaffinity, microdevices, liquid biopsy, ligand-targeted polymerase chain reaction (LT-PCR)

## Abstract

Circulating tumor cells (CTCs) are cells that have been shed from tumors and circulate in the bloodstream. These cells can also be responsible for further metastases and the spread of cancer. Taking a closer look and analyzing CTCs through what has come to be known as “liquid biopsy” has immense potential to further researchers’ understanding of cancer biology. However, CTCs are very sparse and are therefore difficult to detect and capture. To combat this issue, researchers have attempted to create devices, assays, and further techniques to successfully isolate CTCs for analysis. In this work, new and existing biosensing techniques for CTC isolation, detection, and release/detachment are discussed and compared to evaluate their efficacy, specificity, and cost. Here, we specifically aim to evaluate and identify the potential success of these techniques and devices in point-of-care (POC) settings.

## 1. Introduction

**Figure 1:**
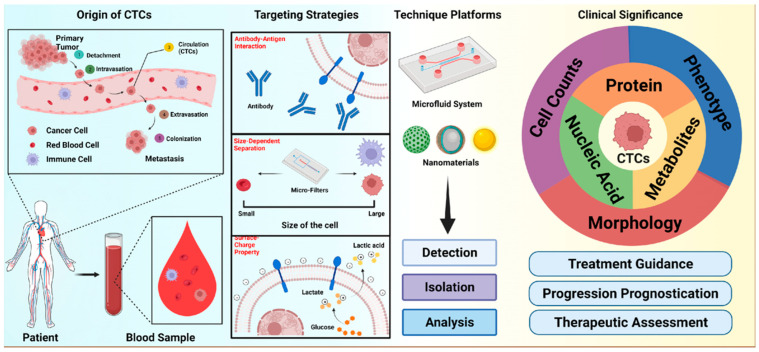
Schematic illustration of circulating tumor cells (CTCs) invasion in the bloodstream and the isolation, detection, analysis, and clinical significance of CTCs. Reprinted with permission from [7].

Currently, the work of many research oncologists centers around a novel targeted-therapy approach to cancer treatment. In order to complete something of that nature, knowing important information about the tumor and cancer is key. After being shed from a primary tumor into the bloodstream, CTCs may provide this key information via a “liquid biopsy”. The process by which CTCs break from a tumor and cause further metastases after traveling in the bloodstream is displayed and described in Figure 1. Since their discovery in 1869 by Thomas Ashworth, the development of CTC research has increased tremendously [1]. Due to their importance in cancer research, many attempts have been made to detect and isolate these cells. Unfortunately, there are many issues that need to be overcome. First and foremost, CTCs are very heterogeneous, meaning that the size, shape, deformability, antibody expression, etc., differs from cell to cell. This makes it extremely challenging to come up with a method that completely encapsulates all CTC properties for complete capture. Furthermore, CTCs undergo epithelial to mesenchymal transition (EMT), which changes many of their properties, and thus the ability to effectively isolate these cells. Moreover, CTCs are extremely sparse, with some estimates numbering CTC presence at one per every 10^9^ hematologic cells in the bloodstream of cancer patients [2]. Despite these obstacles, significant progress has occurred, and there exists one FDA approved device for CTC detection: CellSearch. This method uses anti-EpCAM immunoaffinity (with magnetic nanoparticles) and antibody-linked staining to enrich a sample and detect CTCs. Immunoaffinity, which employs cell-specific antigen identification as a detection mechanism, was an important breakthrough in CTC detection techniques. However, devices that utilize surface epithelial markers suffer from low specificity. To address this limitation, a recent study by Chen et al. demonstrated a digital droplet-microfluidic-flow-cytometry-based biosensor that relies on the in situ detection of dual miRNAs for CTC detection [3]. Conventionally, microfluidic devices that use techniques such as flow cytometry and cell sorting to detect and isolate CTCs have been of key interest in this area. These microdevices use specifically designed precise channels and chambers to capture CTCs from an enriched bloodstream for analysis. The major benefit of this wave of microfluidic innovation is the reproducibility and portability of such devices, which makes them prime candidates for CTC analysis in point-of-care testing applications [4]. However, screening a large number of blood cells to detect and identify CTCs entails low throughput (high processing time) which would be a glaring drawback for the POC testing applicability of such microfluidic devices. The advancement in fabrication technology has enabled microfluidic cytometers and cytometer-adjacent devices to achieve parallelization (processing multiple samples in parallel), and miniaturization (lowering the volume requirement of samples) that add to their point-of-care (POC) applicability [5,6]. In this work, we assess the pros and cons of many of these microfluidic devices in the context of comparing existing detection schemes to recent innovations in an attempt to understand which technologies might be most well-suited for point-of-care testing applications. Although existing methods are useful, improvements concerning cell capture, invasiveness, and efficiency are still necessary.

We make note of the fact that many of the devices, despite showing enhanced potential and exhibiting significant CTC detection and isolation efficiency as proof of concept, may remain incapable of clinical use until further adapted and tested. Beyond providing a comparison of existing and novel CTC detection and isolation techniques, the goal of this paper is to identify the point-of-care potential of the studied devices/assays. As such, in addition to standard measures of success (efficiency, purity, viability, etc.), we value and emphasized non-biological factors such as cost, reproducibility, and portability. In furthering the field of CTC-detection-based cancer diagnosis, this study aims to contribute to the development of effective technology that can be applied outside of large institutional settings and infrastructures and thus improve the ease of diagnosis and disease monitoring in POC settings.

## 2. CTC Isolation Techniques

CTC isolation or enrichment makes subsequent detection much easier. Because CTCs in blood are present in extremely low numbers, isolation techniques aim to “enrich” a sample by increasing the proportion of CTCs to other blood cells. The heterogeneous identity of CTCs further complicates this issue. Though attempted, finding a “one size fits all” approach to CTC isolation is very difficult due to their diverse characteristics (size, shape, antigen expression, etc.). In recent experimentation with isolation techniques, two major approaches have surfaced for CTC isolation: immunoaffinity and physical characteristic isolation. While many techniques fall into either of these two categories, some methods have combined the two, or attempted alternatives. Microfluidic technology has evolved as one of the most popular media for CTC isolation, as microfluidics generally are very cost-effective and easy to use [8].

### 2.1. Immunoaffinity

The most widely used method of CTC isolation, immunoaffinity, works by using antibodies to bind to specific antigens present on cells in order to either target or weed out those cells from a sample. Positive immunoaffinity works by targeting antigens present on CTCs in order to create a filtered sample with more CTCs, whereas negative immunoaffinity is the converse; it discriminates by targeting antibodies present on normal blood cells present in the sample. To overcome the limitations of CellSearch, many technologies have taken its anti-EpCAM-targeting approach and conjugated it with another aspect of CTC isolation. A dual-immunopatterned microfluidic device maintained anti-EpCAM selection in addition to the use of an artificially developed anti-63B6 antibody [9]. The anti-63B6 antibody was able to target mesenchymal-stem-cell-like cancer cells and intermediate cancer cells, a population that is often omitted in strictly anti-EpCAM selection, thus increasing capture efficiency. Manipulation of device shape has often been used as well. A wavy herringbone pattern was implemented in one device that used anti-EpCAM selection with magnetic beads in order to aid the isolation process [10]. The device proved successful in increasing capture efficiency and purity but was limited by the unequal dispersion of magnetic beads and purely anti-EpCAM selection. Similarly, a spiral-shaped microfluidic channel that targeted anti-EpCAM-expressing CTCs with magnetic beads used a purposefully shaped channel in addition to magnetic force as a tool to increase the capture efficiency and flow rate, yet faced the same issue with regard to non-anti-EpCAM-expressing CTC capture [11]. To increase the purity, one method used platelet–leukocyte-membrane-coated immunomagnetic beads [12]. The beads inherited the ability of platelets to interact and assist binding to CTCs in addition to the capability of the leukocytes to reduce homologous leukocyte interaction. This added to the purity, but the applicability of any overall method used with the beads is still hindered by anti-EpCAM capture limitations. While these devices generally display increased capture efficiency as compared to purely anti-EpCAM-targeting methods, they often require greater setup or cost and still have difficulty with non-EpCAM-expressing CTCs. One attempt to curb this problem implemented the use of beads coated with the malaria rVAR2 protein to bind with oncofetal chondroitin sulfate, which is expressed on both epithelial and mesenchymal CTCs [13]. Though a high sensitivity and capture of both epithelial and mesenchymal cells was achieved, a similar problem to anti-EpCAM selection was posed where not all CTCs have strong, or any, expression of oncofetal chondroitin sulfate. Therein lies the flaw in immunoaffinity as a sole solution.

### 2.2. Purely Physical Cell Characteristics

**Figure 2:**
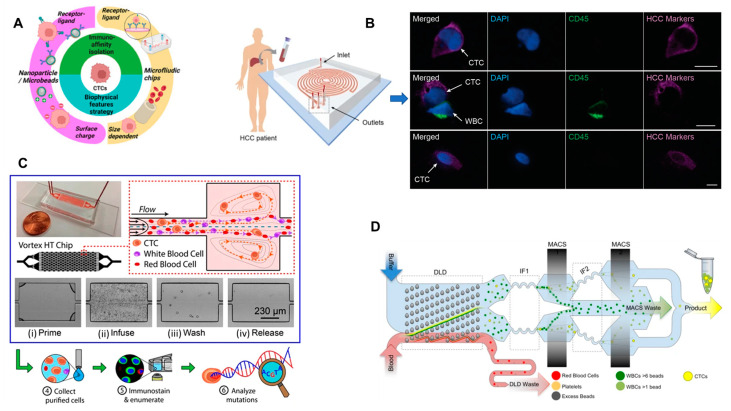
(**A**) Schematic demonstration of different isolation techniques for circulating tumor cells (CTCs). Adapted and reprinted with permission from [7]. (**B**) Labyrinth microfluidic device for the identification and quantification of CTCs isolated from cancer patients. Adapted and reprinted with permission from [14]. (**C**) Microfluidic Vortex HT chip for the isolation and detection of CTCs. Adapted and reprinted with permission from [15]. (**D**) Schematic representation of the monolithic CTC-iChip for the isolation of CTCs from the blood sample. Reprinted with permission from [19].

To avoid the restrictions of immunoaffinity, namely the inability to target cells that lack expression of a particular antigen, many recent approaches to CTC isolation have shifted towards the use of physical cell characteristics. The most common of these characteristics is cell size. Compared to normal blood cells, CTCs tend to have larger diameters, which makes size-based discrimination a useful tool for enrichment. With microfluidic technologies, these methods require the patternization and structure of the device. A so-called Labyrinth device (Figure 2B) was created that achieved a capture efficiency of greater than 90% when using blood samples from breast and pancreatic cancer patients [14]. The purity was also very high, as the device’s strategically placed loops and curves with differing diameters were able to separate CTCs from leukocytes and other blood cells based on their inertial movement due to size/deformability. Another approach used the strategy of cell flow to discriminate based on size. This Vortex HT (high throughput) chip created microvortices on the chip’s surface [15] shown in Figure 2C. When blood samples from prostate cancer were siphoned through, the CTCs would react differently upon reaching the vortices due to their larger size and would be separated from the rest of the sample. Though this device had an extremely high throughput, the efficiency was lower than similar technologies. A largely untapped problem of isolating CTC clusters as opposed to only single cells was resolved by a cluster-isolating device that used deterministic lateral displacement and a microfluidic panel with meticulously spaced micropillars to discriminate clusters and single cell CTCs from a spiked blood sample based on asymmetry and size [16]. This method, while displaying the rare ability to isolate clusters successfully and maintaining cell viability, has a comparatively low throughput, which limits the possibility of clinical use. Other technologies are specifically targeted towards simplicity in a clinical setting. One photosensitive polymer-based microfilter is able to be attached directly to a blood sample obtained by a conventional syringe [17]. This approach uses a polymer that degrades upon contact with UV radiation, revealing a surface with evenly spaced slits, which decrease in diameter towards the bottom of the device. The sample travels through, and the large CTCs are trapped towards the surface. The photosensitive polymer-based microfilter was able to achieve higher efficiency with tested DLD-1G cancer cells than A549G cancer cells, as the former had a much larger average diameter. The CROSS chip was also developed for easy application to normally obtained blood samples without pre-processing [18]. This device uses size and deformability in four different sections to filter larger CTCs from normal blood cells. In a comparative study using blood from metastatic colorectal cancer patients, the CROSS chip performed better in capture efficiency as compared to CellSearch. However, the system lacks the capacity for higher volumes and often needs the tested sample to be screened multiple times for ideal results.

### 2.3. Other Isolation Techniques

While purely immunoaffinity or size-based technology tend to be more common, some researchers have attempted to use a combination of these or entirely different factors for the process of isolation. The SDI (size-dictated immunocapture) chip, combines these two methods in a joint manner [20]. On the surface of the chip, a size-based micropillar approach is implemented, but the micropillars are coated with anti-EpCAM antibodies to direct the flow and capture of the CTCs. This method achieved a 92% capture efficiency and higher sensitivity than CellSearch but faced difficulty in protecting the cells from shear stress during the process. The Monolithic CTC iChip was able to use negative immunoaffinity and size-based capture to discriminate a spiked blood sample with one experiment yielding a median recovery of ~99% [19]. This chip held 128 multiplexed deterministic lateral displacement devices containing ~1.5 million microfabricated features to deplete red blood cells and platelets and used coated magnetic beads to target white blood cells with CD45, CD16, and CD66B surface antigens. However, the device’s inability to filter smaller cells and struggle to remove white blood cells with low expression of the CD45, CD16, and CD66B surface antigens detracts from its extremely high capture rate in previous tests. A lateral filter array was created as well, and used with positive anti-EpCAM discrimination [21]. This device isolated CTCs by using hydrodynamic force with immunoaffinity. If the binding force of the cells (with anti-EpCAM antibodies) was stronger than the experienced hydrodynamic force, the cells would be filtered for isolation. This method achieved a maximum efficiency of 98%, which decreased as the throughput was increased. Though estimates show that the addition of the hydrodynamic filter to normal immunoaffinity techniques added about 10% efficiency, the device still struggles to isolate non-anti-EpCAM-expressing CTCs. The CaTCh FISH chip is another device that uses negative immunoaffinity [22]. The first step of the two-step process includes tagging CD45-expressing white blood cells with magnetic nanoparticles. Specially placed magnetic micropores then attract these cells into traps before the blood sample is subjected to a size-based filter. A spiked sample was sorted with 90% efficiency using this method, but once again, the difficulty of removing white blood cells with low CD45 expression rates is a significant issue. LFFF-DEP (lateral fluid flow fractionation-dielectrophoresis) technology is a method that uses the difference in conductivity between CTCs and normal blood cells to discriminate a sample [23]. The CTCs will experience positive dielectrophoresis and migrate towards one electrode, while the rest of the sample will typically experience negative dielectrophoresis. Though no statistical analysis was conducted with this technology, a test trial with breast cancer patient blood was described as “successful”. Another innovative approach uses optofluidic technology to enrich a sample [24]. This approach uses folic acid to facilitate the binding of multiple homologous red blood cells with CTCs. This increases the refractive index of the much larger CTC-RBC combination cells and allows for laser illumination to be used as a separation tool before the red blood cells are lysed. This approach demonstrated a highly successful recovery rate of ~90% and a purity of 92%, although the cost of such a process is unspecified. Acoustic separation has been another area of study with regard to CTC isolation. One recent acoustic separation technique used tilted angle standing surface acoustic waves in part with a polydimethylsiloxane-glass channel to form an acoustic enclosure, thus boosting the energy density of the acoustic waves [25]. Once a sample was filtered through the chamber, the acoustic waves would change the trajectory of the cells dependent on their size/shape and separate the leukocytes from the CTCs. Though this process achieved 86% capture efficiency and a high throughput of 7.5 mL/h, it requires a pre-processing RBC lysing step, which increases the operational time and may mistakenly remove CTCs from the sample. Table 1 provides a comparative summary of all these isolation techniques.

## 3. CTC Detachment/Release Techniques

Because the ultimate goal is to analyze CTCs to help with cancer research, the process does not end with detection and isolation. Detachment from surfaces is another step that requires different techniques. After cell capture, CTCs must be released in an efficient manner, yet one that still allows the cells to remain intact and retain viability so that they can be cultured and analyzed. Aptamers, nanodevices, and less conventional methods involving light, electric, and chemical processes have been used for CTC detachment.

### 3.1. Aptamers

**Figure 3:**
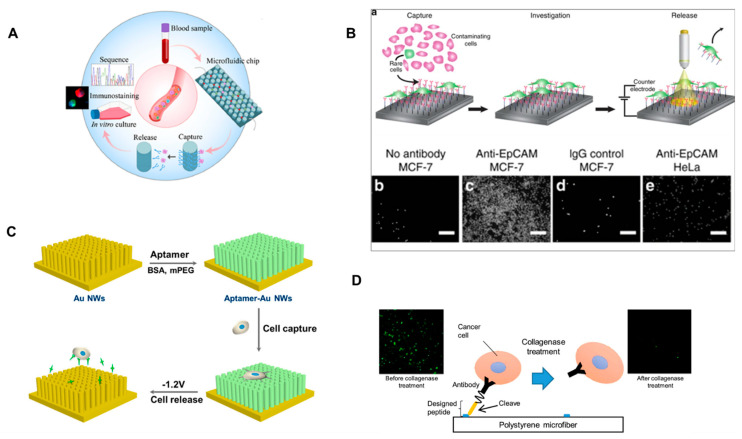
(**A**) Process flow of CTCs’ capture, release, and downstream molecular analysis. Adapted and reprinted with permission from [7]. (**B**) Photoelectrochemical platform for capturing and releasing CTCs on antibody-modified surfaces. Adapted and reprinted with permission from [33]. (**C**) Aptamer-modified gold nanowires (AuNWs) for capturing and releasing CTCs. Reprinted with permission from [29]. (**D**) Polymeric microfibers employ enzyme-cleavable peptides to release captured cancer cells. Reprinted with permission from [31].

The use of aptamers has been one of the most common methods in CTC detachment as of late. These oligonucleotide or peptide molecules have impressive binding capability, which can be manipulated for cell release by altering their tertiary structures. Aptamers are known to be highly selective and sensitive while these withstand unfavorable surroundings/conditions [26,27]. This process has been used in many devices such as the previously developed NanoVelcro chip, where 85% of CTCs were released, and around 80% maintained viability after surface-grafted aptamers were cleaved using Benzonase Nuclease [28]. Other devices have built upon this aptamer-based approach and received more successful results. Aptamer-modified gold nanowires used aptamer-sgc8c (Figure 3C). After isolated CTCs bonded to the surface, electrochemical reduction desorption at −1.2 volts for 30 s was used to cleave gold–sulfur bonds (which are only stable under “normal” conditions), thus releasing the CTCs. This method achieved a 96.2% release efficiency in addition to a 90% post-release viability rate [29].

### 3.2. Microdevices/Nanodevices

Nanotechnology and microtechnology have also been prominent within the realm of CTC detachment. Nanoparticles in particular have been commonly used to facilitate successful and easy release. One study used gold nanoparticles conjugated with a mixed monolayer of 11-mercaptoundecanoic acid (MUA) and 12-mercaptododecanoic acid N-hydroxysuccinimide ester (NHS). The NHS ligands bound an amine moiety to NeutrAvidin, which held the CTCs in place on the surface of a microchip. Glutathione was then used to cleave the bonds, as it is easy to come by and use. This process resulted in release efficiencies of 92% and 91% and cell viabilities of 87% and 78% for the isolated PC3 and MDA-MB-231 cancer cells, respectively [30]. Another device used polymeric microfibers implemented with an anti-EpCAM cell isolation approach (Figure 3D). A base of polystyrene microfibers was constructed, and specifically selected peptides with an anti-EpCAM antibody on one end were bound to the base. These peptides were cleavable by collagenase type IV, which resulted in a cell release efficiency of over 90% and a viability of 83% [31]. Though successful, the technology is nevertheless restricted by the same hindrances as all anti-EpCAM selection techniques. One process with an exceedingly high release rate of about 95% is the use of biodegradable nano-films. These nano-films are presented layer-by-layer with anionic and cationic polymer solutions and are conjugated with antibodies for cell capture (most commonly anti-EpCAM). An enzymatic solution is used to induce degradation of the nano-films, resulting in cell release. In an experimental study with spiked prostate cancer cell samples, the cells retained 90% viability after release [32].

### 3.3. Light/Electrochemical

Additional detachment techniques that have gained interest involve light and electrochemical release. These methods are favorable due to their minimally invasive nature yet may involve higher costs or an increased setup time. One tried detachment process is photoelectrochemical single cell release, which is beneficial in that it is able to target single cells, as opposed to the only detachment option being mass release. An experiment with this method utilizes carefully placed semi-conducting electrodes; when light is shined, electrons in the conduction band of the silicon surface are excited, increasing conductivity which prompts cleavage and single cell release (Figure 3B). At −1.2 volts for 240 s, the release was somewhat successful at around 82%, while the viability was also higher at around 90% [33]. A second light-based approach used a light-responsive hydrogel. Artificial anti-EpCAM receptors were imprinted on the gel base, which was also embedded with gold nanorods. Once CTCs bind to the artificial receptors, selected locations on the gel are exposed to near-infrared radiation (NIR). The gold nanorods heat up as a result of this process (dubbed photothermal activation), causing the gelatin to dissolve, releasing roughly 92% of all captured MCF-7 cells, which maintain 90% viability. Though this process can target specific cell release, a bulk-release approach in which the gel was simply heated to 37 °C maintained higher release and viability rates of 95% each [34]. Table 2 illustrates the detachment techniques with their merits.

## 4. CTC Detection Techniques

The detection of CTCs is integral to the concept of “liquid biopsy”. Being able to determine whether CTCs are present in a blood sample and how significant of a presence they have is key information for diagnostic and therapeutic decision-making for oncologists. In order to provide a systematic review of the CTC detection schemes, a comprehensive analysis of various methods and technologies used for the detection and counting of CTCs from blood samples was performed. After a comprehensive literature search and screening based on the predetermined criteria, most of the conventional assays and advanced detection methods were analyzed and compared for their performance characteristics. This analysis included a critical evaluation of the advantages and limitations of each method, as well as their sensitivity, specificity, reliability, need for fluorescent labeling, complexity, and cost. A systematic review of these schemes showed that they can be broadly classified into two major categories: direct detection, which occurs without enrichment, and post-enrichment detection methods. CellSearch, the FDA-approved system, falls under the category of a post-enrichment detection system that uses immunoaffinity (anti-EpCAM) to first enrich the sample and then employs antibody-linked staining to detect the CTCs within the sample.

### 4.1. Direct Detection (Pre-Enrichment)

Direct detection techniques hold a significant advantage over post-enrichment techniques. Because enrichment is not a necessary step, the process becomes much simpler and often requires less time and effort concerning the pre-processing steps. However, these techniques also tend to be on the higher end with regard to cost and may be slightly more difficult to implement effectively. One of these direct detection strategies is SERS (surface-enhanced Raman scattering) detection using active magnetic nanoparticles [35,36]. With this strategy, magnetic nanoparticles are conjugated with folic acid, and the folate receptor on CTCs allows for binding. This allows for CTCs to display a much higher SERS intensity than normal cells, which in turn causes a greater signal within the blood that undergoes Raman scattering analysis. In one experiment with HeLa cells, this method was able to detect anywhere from 4–18 CTCs per 10 mL of blood [37]. Another direct investigation of CTCs was reported using solid-state micropores. This approach was able to detect CTCs one cell at a time, and electronic fingerprinting was recorded to identify and count the CTCs [38,39]. This highly sensitive technique reported more than 90% accuracy and parallel microchannels for high throughput [6]. Another direct detection possibility is the use of a photoacoustic cytophone. This in vivo testing method, though promising, is specific to melanoma. It works via the laser heating of light-absorbing hemoglobin in red blood cells and melanin expressed in melanoma CTCs, which causes the thermoacoustic and nanobubble-based generation of acoustic waves. These waves can be detected by a small ultrasound transducer placed over the skin, which displays a large spike when CTCs flow by, and a negative spike when white blood cells and platelets flow by. This method was able to detect individual CTCs at a concentration of ≥1 CTC per mL in 20 s and had an estimated specificity of 95% [40]. An optical method for direct detection known as confocal microscopy has also proved successful, although it is quite expensive. This method requires fluorescent labeling and uses a laser microscopy technique (originally based on inverted microscopy) to detect the fluorescently labeled CTCs. In one experiment the human hepatocellular carcinoma cell line (HCCLM3) was injected with enhanced green fluorescent protein into mice. Confocal microscopy was able to distinguish single CTCs from clusters, even at relatively early stages, and by day 30, the CTC counts numbered as high as 45.4 ± 6.2 per hour [41].

### 4.2. Post-Enrichment Detection

More common methods for detection are post-enrichment, as an enriched sample has a much higher proportion of CTCs, which makes them easier to detect. As discussed earlier, the CaTCh FISH chip is used for isolation/enrichment, but it can also be used for post-enrichment detection. The technology uses nucleic acid detection. Specifically, direct labeling occurs via RNA fluorescence in situ hybridization (RNA FISH), which involves the hybridization of 20–50 short, fluorescently labeled oligonucleotide probes to the target RNA by Watson–Crick base pairing. The fluorescence is then used as a marker, as it will appear more intense in spots with CTCs [22]. The EPISPOT (epithelial immunospot assay) is a test that in theory can be attached to any enrichment step. Membranes are coated with antibodies against specific protein markers, and secreted proteins are directly captured on the antibody-coated membrane. When samples are incubated with the membranes, only viable captured CTCs will be able to capture the proteins, which are then used to direct fluorescence. The end result will be increased fluorescence in the areas with viable CTCs, which is helpful in discriminating against apoptotic/unviable CTCs [42]. Immunonanospheres, or nanosphere detection, is one process that allows for simultaneous detection and isolation. The nanospheres are conjugated with green fluorescence and an anti-EpCAM antibody to target CTCs and red fluorescence and an anti-CD45 antibody to target white blood cells. The nanospheres’ magnetic properties also allowed for enrichment with up to 96% efficiency [43]. A similar study used fluorescent magnetic beads (a combination of anti-EpCAM, anti-EGFR, and anti- VMT beads used) with a parallel flow micro-aperture chip, and they were able to detect CTCs with 89% efficiency. This device also contained a strong magnetic field and size filters to help with enrichment and detection [44]. The GILUPI CellCollector was a device built to contest the ability of CellSearch. The CellCollector device can be directly inserted through a catheter into a patient’s vein. The system works by immunoaffinity fluorescent staining using both anti-CD45 to discriminate against white blood cells and anti-EpCAM to discriminate for CTCs. When studied using colorectal cancer patients, the CellCollector system showed no significant improvement over CellSearch and was deemed to have lesser “clinical relevance” [45]. Additionally, an LT-PCR (ligand-targeted polymerase chain reaction)-based method was used in an experiment with lung cancer patients. Blood samples were first enriched by immunomagnetic depletion of white blood cells. Folate-receptor-positive CTCs were then targeted for detection using a conjugate of a tumor-specific ligand folic acid and a synthesized oligonucleotide. Once bound, PCR quantification was able to detect the CTCs due to the presence of the oligonucleotide. This process demonstrated a sensitivity of 74.4% and a specificity of 86.6% [46]. Table 3 summarized the CTCs detection techniques.

## 5. Conclusions

Liquid biopsy has proven to be an extremely important tool for cancer research. It has functions not only in cancer identification and monitoring but may lead to new therapeutics and treatments. Though this idea holds much promise, certain barriers still need to be overcome. The heterogeneity of CTCs makes it extremely difficult to find an approach that detects or isolates almost all of the given CTCs in a sample, and cell viability is preserved for downstream analysis when attachment occurs. Though these aforementioned technologies are certainly encouraging, more research and testing are still necessary in this field to find methods that are both cost-efficient and easy to use, while achieving high sensitivity, selectivity, detection efficiency, and portability to meet the requirements of a POC testing device.

Furthermore, there is a persistent gap between proofs of concept and real devices that have been approved for clinical use. Many of these promising technologies for CTC detection and isolation have performed well strictly in a laboratory setting. Beyond the FDA approval of CellSearch, most of these assays/devices must overcome additional performance tests and regulatory barriers before they can be clinically used for point-of-care testing applications.

## Figures and Tables

**Table 1:** CTCs Isolation Comparison Chart.

Technology	Isolation Factors	Benefits	Drawbacks	Description	Reference
CROSS Chip (Microfluidic Cell Filter)	Size and deformability	70% efficiency, high purity, cost-effective, easily applicable (low set-up), and higher sensitivity than CellSearch	To increase throughput, multiple screenings might be necessary; smaller CTCs are more difficult to obtain	A syringe pumps the blood sample into a microfluidic chip with filters that separates it into four sections for analysis	[18]
Labyrinth (Inertial Microfluidic Cell Filter)	Size (inertia)	High throughput, high yield, and high purity	Difficulty with focusing and separating smaller cells	The labyrinth design functions in separating CTCs from blood cells by filtering them through straight and curved channels	[14]
Optofluidic Cell Technology Chip	Size and the refractive index	High purity, high recovery, and no foreign material introduction	Time and cost unspecified	Uses molecules to bind CTCs to RBCs and then uses laser illumination to separate them on a chip, based on the refractive index	[24]
Dual-Immunopatterned microfluidic device	Surface antigen expression	High efficiency and captures mesenchymal cells	More time, possibly experiment-dependent, and less general applicability	Microfluidic device with two layers. Each layer is coated with a different antibody (anti-EpCAM or anti-63B6) to isolate and separate the types of CTCs	[9]
Acoustic Separation Device	Cell size/velocity (response to sound waves)	High throughput and cost-effective	Prior processes required for RBC removal and more time	The device uses the acoustic-wave field that is amplified by a PDMS barrier to send cells at different trajectories for separation	[25]
Wavy-Herringbone Microfluidic Device	Immunoaffinity (+EpCAM) and magnetic force	High efficiency, high purity, and high viability	Dependent on the dispersion of MPs (hard to control) and may be unable to capture non-EpCAM-expressing CTCs	A herringbone pattern was made on silicon wafers which were injected with bonded magnetic particles and anti-EpCAM. As the sample travels through, the CTCs are separated by the magnetic EpCAM	[10]
Vortex HT Chip	Size (movement)	Extremely high throughput, high viability, high purity, and no pre-processing steps	Certain CTCs not captured effectively, possible difficulties with cell recovery, and low capture efficiency	Laminar microvortices are created on this microdevice to separate CTCs from blood cells and other bodily fluids based on flow	[15]
Lateral filter array with immunoaffinity	Size and immunoaffinity (+EpCAM)	High efficiency, high purity, and high viability	May be unable to capture certain non-EpCAM-expressing CTCs	Embedded lateral filters in a serpentine channel on a microfluidic device. The blood sample flows through the main channel and the CTCs are caught in the filter. Immunoaffinity works in the lateral filters by testing for a bond force between certain antibodies and the cells as compared to the hydrodynamic force	[21]
Spiral Shape Microfluidic Channel	Magnetic force and immunoaffinity (+EpCAM)	High efficiency and high flow rate	May be unable to capture non-EpCAM-expressing CTCs; more tests with actual samples necessary	Magnetic nanoparticles bond with the EpCAM antibody. These are circulated through a spiral chamber with a decreasing radius and a permanent magnet. The magnetic force causes the bonded particles to be attracted and filter into specific regions for isolation	[11]
LFFF-DEP microfluidic device	Dielectrophoresis	No statistical results; however, based on the provided image, it seems to be efficient in separation	Further analysis necessary and time and throughput may be an issue	Oppositely charged electrodes are positioned on a glass wafer. Cancer cells are attracted to the positive DEP electrode, while normal blood cells are attracted to the negative DEP electrode	[23]
Cluster-isolating microfluidic device	Size and asymmetry	Able to isolate clusters, high recovery, and high viability	The flow rate has to be slowed to ensure viability (very low throughput) and less adept to isolating small clusters and single CTCs	Deterministic lateral displacement is used to isolate large clusters based on size (using micropillars with different sized gaps in between). During stage two, the clusters that were unable to be filtered by size are put through shaped micropillars that result in rotation if they are asymmetrical for separation	[16]
Magnetic Micropore CaTCh FISH Chip	Magnetic force (immunoaffinity of -CD45 on WBC) and size	Higher throughput compared to other magnetic chips, High recovery rate and the ability to conduct RNA analysis on the chip	WBC with low CD45 expression not filtered and cost unclear	Two-part system. The first section of the chip has magnetic traps at edges of the pores to attract WBCs labeled with MNPs. The RBCs and platelets are then filtered by size leaving the CTCs, which undergo RNA analysis on the chip	[22]
rVAR2 using IsoFlux system	Immunoaffinity (+ofCS)	High recovery, captures mesenchymal cells, and high sensitivity	May be unable to capture non-ofCS-expressing CTCs and may be expensive	Uses the IsoFlux system model (Dynabeads) but altered so the immunomagnetic capture is used with the rVAR2 protein to bond to the ofCS in the CTCs	[13]
PLT-WBC Immunomagnetic Beads	Immunoaffinity (+EpCAM) and magnetic force	High Efficiency, increased binding ability, and avoidance of WBC collection	May be unable to capture non-EpCAM-expressing CTCs and requires additional preparation process for magnetic beads	A hybrid membrane of platelets and WBCs is formed and coated onto magnetic particles. The particles are then treated with EpCAM-binding antibodies. The resulting magnetic beads target the CTCs while specifically avoiding homologus WBCs	[12]
Photosensitive Polymer-Based Microfilter	Size and deformability	High efficiency, relatively high viability, and simple set-up	Smaller CTCs are difficult to capture	The photosensitive polymer which is removed with UV exposure coats a microfilter with many densely dispersed slits. The slits have a larger inlet which decreases in diameter towards the outlet, trapping larger CTCs	[17]
SDI Chip	Immunoaffinity (+EpCAM) and size	High efficiency, high purity, and greater sensitivity as compared to CellSearch	Lower-expression EpCAM was more frequently not recovered and shear stress caused the dislodgement of many cells	Microchip on which triangular micropillars are coated with anti-EpCAM antibodies. The pillars are spaced and rotated to create a decreasing hydrodynamic gradient and gaps. Due to gradient cells migrating downstream and at certain locations due to their size and immunoaffinity, the CTCs become lodged at pillars	[20]
Monolithic CTC iChip	Immunoaffinity (-CD45, -CD16, and -CD66B) and size	High throughput and high recovery	Smaller cells had difficulty being caught, WBCs with low antigen expression levels and difficulty being caught, and average purity	The microfluidic chip first holds a size-based array with micropillars with a waste channel for RBCs and plasma. The next portion has magnets on either side. The magnetic-tagged WBCs are filtered to the sides, while the CTCs are caught in the middle (non-magnetic) portion.	[19]

**Table 2:** CTC detachment comparison chart.

Technology	Release Mechanism	Viability	Release Efficiency	Reference
Biodegradable Nano-Films	The enzyme solution degrades the polymeric film	~90%	95%	[32]
Aptamer Modified Gold Nanowires	Aptamer sgc8c conjugated with gold nanowires; electrochemical reduction desorption at −1.2 V for 30 s breaks the Au–S bonds	90%	96.2%	[29]
Gold Nanoparticles	NHS ligands bond the amine moiety to NeutrAvidin; glutathione is used to break bonds	91.5% (average of two tests with MDA-MB-231 and PC3)	82.5% (average of two tests with MDA-MB-231 and PC3)	[30]
Polymeric Microfibers	Peptides are bound to the polystyrene microfiber and anti-EpCAM antibody; collagenase type IV is used to cleave the peptide	83%	>90%	[31]
Photoelectrochemical Single Cell Release	Light activates the electrons on a conduction band on a silicon surface; it increases the conductivity which prompts cleavage and single cell release	90 ± 2.7%	82 ± 5%	[33]
Light-Responsive Hydrogel	The near infrared-mediated photothermal activation of embedded gold nanorods causes temperature-responsive gelatin to dissolve rapidly at 37 °C	95 ± 4% (overall thermal release rate) 92 ± 6% (release rate using specified light beams/photothermal selection)	95 (overall thermal viability rate) 90% (viability rate using specified light beams/photothermal selection)	[34]

**Table 3:** CTC detection comparison chart.

Technology	Labeling	Cost Description	Beneficial Qualities	Includes an Enrichment Step
CellSearch	Yes	USD 350	FDA-approved	Yes
SERS active magnetic nanoparticles	No	Expensive equipment needed	High efficiency and high purity	No
Solid-state Micropores	No	Low cost	High sensitivity	No
Photoacoustic Cytophone	No	Low cost	High specificity	No
CaTCh FISH Chip	Yes	Costly	High sensitivity	Yes
EPISPOT Assay	Yes	USD 300–400 based on previous related assay costs	Simple and easily applicable	Yes
Nanosphere Detection	Yes		High efficiency and cell viability post enrichment	Yes
GILUPI CellCollector	Yes	Described alternative methods as “costly”	Relatively high sensitivity	Yes
Parallel Flow Micro-Aperture Chip	Yes		High detection yield	Yes
LT-PCR Based Method	Yes	Cost effective	Medium/high sensitivity and specificity	Yes
Confocal Microscopy	Yes	Very expensive (the product that served as the basis is USD 10k, but estimates range up to USD 100k+)	Extremely simple, no necessary adjustments	No

## Data Availability

The data presented in this study are available upon request from the corresponding authors.
